# Effects of aging and involuntary capture of attention on event-related potentials associated with the processing of and the response to a target stimulus

**DOI:** 10.3389/fnhum.2014.00745

**Published:** 2014-09-23

**Authors:** Susana Cid-Fernández, Mónica Lindín, Fernando Díaz

**Affiliations:** Laboratorio de Psicofisioloxía e Neurociencia Cognitiva, Departamento de Psicoloxía Clínica e Psicobioloxía, Facultade de Psicoloxía, Universidade de Santiago de Compostela, Santiago de Compostela, A CoruñaGaliza, Spain

**Keywords:** involuntary capture of attention, distraction, aging, event-related potentials, N2b, P3b, lateralized-readiness potential (LRP)

## Abstract

The main aim of the present study was to assess whether aging modulates the effects of involuntary capture of attention by novel stimuli on performance, and on event-related potentials (ERPs) associated with target processing (N2b and P3b) and subsequent response processes (stimulus-locked Lateralized Readiness Potential -sLRP- and response-locked Lateralized Readiness Potential -rLRP-). An auditory-visual distraction-attention task was performed by 77 healthy participants, divided into three age groups (Young: 21–29, Middle-aged: 51–64, Old: 65–84 years old). Participants were asked to attend to visual stimuli and to ignore auditory stimuli. Aging was associated with slowed reaction times, target stimulus processing in working memory (WM, longer N2b and P3b latencies) and selection and preparation of the motor response (longer sLRP and earlier rLRP onset latencies). In the novel relative to the standard condition we observed, in the three age groups: (1) a distraction effect, reflected in a slowing of reaction times, of stimuli categorization in WM (longer P3b latency), and of motor response selection (longer sLRP onset latency); (2) a facilitation effect on response preparation (later rLRP onset latency), and (3) an increase in arousal (larger amplitudes of all ERPs evaluated, except for N2b amplitude in the Old group). A distraction effect on the stimulus evaluation processes (longer N2b latency) were also observed, but only in middle-aged and old participants, indicating that the attentional capture slows the stimulus evaluation in WM from early ages (from 50 years onwards, without differences between middle-age and older adults), but not in young adults.

## Introduction

In everyday situations, we often need to focus on a task, while ignoring irrelevant events occurring around us. However, certain unexpected events sometimes interfere with the focus of our conscious evaluation, leading to involuntary capture of our attention. This involuntary attentional shift is known as the orienting reflex (Sokolov, 1990), and it is very important for survival under natural conditions. However, these rare, salient stimuli often distract from the primary task (distraction effect), which can lead to less efficient processing of the task-relevant stimuli (Escera et al., 1998, 2001; but see SanMiguel et al., 2010), and also less efficient performance (Escera et al., 2001; Parmentier et al., 2008, 2011; but see Wetzel et al. (2011), for a detailed evaluation of the distraction effect).

Several studies have evaluated, in young and old adults, the effect of involuntary capture of attention on relevant stimuli processing during the performance of auditory duration discrimination tasks and dichotic-listening distraction tasks (Gaeta et al., 2001; Mager et al., 2005; Horváth et al., 2009; Berti et al., 2012). In these studies, participants were required to respond to one feature of the auditory stimuli (tonal duration) while another irrelevant feature served as a distractor (change in the tonal frequency). Reaction times (RTs) in response to the distractor condition were longer when compared to the non-distractor condition. In one of these studies, this effect was significantly larger in the older than in the younger group (Gaeta et al., 2001), while in the others it did not differ significantly between age groups (Mager et al., 2005; Horváth et al., 2009; Berti et al., 2012). Some of these studies also reported less correct responses (hits) in the distractor condition, in both age groups (Berti et al., 2012), while others did not find any such difference (Mager et al., 2005).

Another task used by some researchers with the same aim as above, was an auditory-visual distraction-attention task (Andrés et al., 2006; Parmentier and Andrés, 2010), adapted from Escera et al. (1998, 2001). Both studies observed longer RTs (in response to visual stimuli) in both young and elderly participants in the novel condition (when the target visual stimulus was preceded by a novel auditory stimulus) than in the standard condition (when the target visual stimulus was preceded by a standard auditory tone). But, while Parmentier and Andrés (2010) didn't observe significant differences between age groups, Andrés et al. (2006) found a significantly stronger effect in the elderly group. The last result was interpreted as reflecting a decline in frontal or anterior attentional networks in the older groups, in which filtering of irrelevant information must be accomplished.

Until now, the auditory-visual distraction-attention task in conjunction with the ERP technique, evaluating the involuntary capture of attention, was only used with young people (Escera et al., 1998, 2001; Yago et al., 2001a,b, 2003; Polo et al., 2003; SanMiguel et al., 2010). Evaluating the novelty effect on the target visual stimuli processing, Escera et al. (1998) found longer RT and larger amplitudes of N2b and P3b ERP components to the target stimuli for the novel condition than for the standard condition. However, SanMiguel et al. (2010) observed shorter RTs and larger P3b amplitudes in response to the target visual stimuli, in the novel than in the standard condition, while the number of correct responses did not differ between conditions. These results were interpreted as a facilitation effect caused by the novel stimuli on target processing, possibly due to a large expectation activity in the visual cortex. The authors suggested that novel sounds resulted in a greater amount of attentional capacity being invested in the posterior categorization of the visual target, and that the facilitation effect by novel sounds can be explained by the arousal component of the orienting response (OR) that they generate (SanMiguel et al., 2010).

The N2b component is a negative wave that appears about the 200–300 ms after the target stimulus presentation in young people (Hämmerer et al., 2010), with maximal amplitudes at central scalp locations in young and old adults (Amenedo and Díaz, 1998a,b). It indicates the first step of conscious sensory discrimination, when active evaluation of the stimulus in working memory (WM) is performed (Ritter et al., 1979). The P3b component is a positive wave with latency about 300–500 ms after target presentation in young people (Escera et al., 2001; Helenius et al., 2010). P3b amplitude is maximal at parietal-central scalp locations in young participants (Anderer et al., 2003), and at central-frontal locations in elderly participants (Fabiani and Friedman, 1995; Amenedo and Díaz, 1998a; O'Connell et al., 2012). It has been related to the amount of neural resources assigned to categorization of the target stimulus (Donchin and Coles, 1988). Moreover, P3b latency has been interpreted as an index of the time required to evaluate and categorize the stimuli in WM (Coles and Rugg, 1996).

The RT indicates the stimulus processing time, as well as the time needed to select, prepare and execute the response. Nevertheless, the ERP technique allows us to evaluate separately, and with a temporal resolution on the order of milliseconds, the characteristics of brain electrical activity associated with the selection and the preparation of the motor response. Hence, we also decided to determine whether the involuntary capture of attention affects the brain electrical activity associated with the selection and preparation of the response to the visual target stimuli, in the three age groups. For this purpose, we examined the lateralized-readiness potential (LRP). The LRP is a negativity computed from the ERP recorded above the hand areas of the motor cortices of both hemispheres (Lehle et al., 2011), and it is considered an indicator of the effector-specific motor response choice (stimulus-locked LRP or sLRP) and of the motor planning (response-locked LRP or rLRP; Roggeveen et al., 2007).

On the other hand, aging is usually associated to latency increases of N2b and P3b and amplitude decreases of P3b (Patel and Azzam, 2005; Polich, 2012), and amplitude and onset latency increases in both sLRP and rLRP (Roggeveen et al., 2007; Wild-Wall et al., 2008; Vallesi and Stuss, 2010). However, the interaction effects between aging and the capture of attention on these components are still unknown. Therefore, in the present study we recorded ERPs in three groups of participants (Young, Middle-aged, and Old) during an auditory-visual distraction-attention task with the following aims:

1. To evaluate the effect of aging on task performance and on the N2b and P3b latencies and amplitudes and the sLRP and rLRP parameters (amplitudes and onset latencies), measured in response to the target visual stimuli. We expected to find an age-related decrease in the percentage of hits as well as a slowing of the RT and of the latencies of the ERPs components evaluated.
2. To evaluate, in each age group, the effect of involuntary capture of attention provoked by the auditory novel vs. standard stimuli on the RT, the percentage of hits and on the N2b, P3b, sLRP, and rLRP parameters, measured in response to the target visual stimuli. We expected to observe in the novel condition relative to the standard condition: (1) an increase in the RT and a decrease in the percentage of hits, (2) longer N2b and P3b latencies, and (3) longer sLRP onset latencies. We expected that this effect would increase with aging, and that it would be stronger in the old and middle-aged adults than in the young participants, and stronger in old than in middle-aged adults.

## Materials and methods

### Participants

In total, 77 healthy participants (52 women; age range: 21–84 years old) participated voluntarily in this study. The participants were divided in three age groups: (1) Young (*N* = 23; 17 women; mean age: 23.5 years, SD: 2.9); (2) Middle-aged (*N* = 26; 15 women; mean age: 57.9 years, SD: 3.5); and (3) Old (*N* = 28; 20 women; mean age: 71.7 years, SD: 4.9). The groups were matched according to level of education [Young: mean = 55.8, *SD* = 6.3; Middle-aged: mean = 53.9, *SD* = 13.3; Old: mean = 51.7, *SD* = 10.3; *F*_(2, 73)_ = 0.94; *p* = 0.394], as assessed by the vocabulary subtest from the Wechsler Adult Intelligence Scale (WAIS; Wechsler, 1988). The young participants were all university students or graduates, except one who had completed compulsory secondary education. The middle-aged and the old adults had no cognitive deficits, as assessed by the Spanish version of the Mini-Mental State Examination (Middle-aged: mean = 28.7, *SD* = 1.0; Old: mean = 27.8, *SD* = 1.8; Folstein et al., 1975; Spanish version by Lobo et al., 1999).

The participants had no history of clinical stroke, traumatic brain injury, motor-sensory deficits, alcohol or drug abuse/dependence, and they were not diagnosed with any significant medical or psychiatric illnesses. All participants had normal audition and normal or corrected-to-normal vision. Most of the participants were right-handed, as assessed by the Edinburgh inventory (Oldfield, 1971), except for one who was left-handed and two who were ambidextrous.

### Procedure

The auditory-visual distraction-attention task used was adapted from Escera et al. (1998, 2001). The task included an auditory passive oddball task and a visual active three-stimulus task. Participants were presented with 500 pairs of auditory-visual (A-V) stimuli, divided into 2 blocks with a short rest between each block. Each pair consisted of a visual stimulus (200 ms duration) preceded by an auditory stimulus (150 ms duration), separated by an interval of 300 ms (SOA), and with an interval of 2 s between each pair. Participants were asked to attend to the visual stimuli and to ignore the auditory stimuli. The task procedure is summarized in Cid-Fernández et al. (2014) (see their Figure 1).

The attended visual stimuli were numbers (2, 4, 6, 8), letters (a, e, c, u) and triangles (pointing upwards, downwards, or to the right or left). Participants were instructed to respond to numbers (33%) and to letters (33%), by pressing a button (Go stimuli; target) with their left hand for one type of stimulus and with the right hand for the other type (the response hand was counterbalanced among participants), and to inhibit their responses to triangles (34%; NoGo stimuli). In this study, only the Go condition was evaluated. The non-attended auditory stimuli comprised 3 types of sounds presented binaurally via headphones at 75 dB SPL; 70% were standard stimuli (1000 Hz pure tones), 15% were deviant stimuli (2000 Hz pure tones), and 15% were novel stimuli (which differed each time, e.g., glass crashing).

### EEG recording

The participants were seated on a comfortable chair in a Faraday chamber, with attenuated levels of light and noise, and were instructed to move as little as possible during the recording. Visual stimuli were presented with a subtended visual angle of 1.7° × 3.3° of arc, on a 19″ flat screen monitor with a vertical refresh rate of 120 Hz. The monitor was located 1 m away from the participant. The EEG was recorded via 49 electrodes placed in an elastic cap (Easycap, GmbH), according to the International 10-10 System. All electrodes were referenced to an electrode attached to the tip of the nose, and an electrode positioned at Fpz served as ground. The horizontal electrooculogram (EOG) was recorded via two electrodes placed at the outer canthi of both eyes, whereas the vertical EOG was recorded via two electrodes placed supra and infraorbitally to the right eye. The EEG was continuously digitized at a rate of 500 Hz (bandpass 0.01–100 Hz), and electrode impedances were maintained below 10 kΩ. Once the signal was stored, ocular artifacts were corrected and the EEG was segmented. Only the epochs associated with the standard auditory-target visual pairs (standard condition) and novel auditory-target visual pairs (novel condition) were evaluated.

With the aim of evaluating the N2b and P3b components, and to obtain the sLRP, the EEG was segmented by extraction of auditory stimulus-locked epochs of 1450 ms (150 ms pre-stimulus). To obtain the rLRP, the EEG was segmented by extraction of response-locked epochs of 1300 ms (1000 ms pre-response and 300 ms post-response). The signal was passed through a digital 0.1–30 Hz (24 dB/octave slope) bandpass filter, and epochs were corrected to the mean voltage of the prestimulus recording period. Segments exceeding ±100 μ V were automatically rejected.

In order to identify N2b and P3b components, the 1450 ms-EEG epochs were averaged separately for the standard and novel stimuli. A minimum of 20 artifact-free epochs were averaged. For the sLRP and rLRP, the epochs (of 1450 ms duration for sLRP and of 1300 ms duration for rLRP) were averaged following two criteria: the type of auditory stimulus (novel or standard) and the hand of response to the visual target (right or left). Thus, four different averages were obtained: novel-right hand, novel-left hand, standard-right hand, and standard-left hand. A minimum of 38 artifact-free epochs were averaged.

Finally, in order to obtain the sLRP and rLRP waveforms, the differences between contralateral and ipsilateral activation for C3 and C4 electrode pairs in each hemisphere were calculated. The differences were then averaged (Gratton et al., 1988). The method can be summarized by the following formula: [[(C4–C3)_left hand movements_ + (C3–C4)_right hand movements_]/2].

### Data analysis

Reaction times (between the onset of the visual stimulus and pressing the key) and the percentage of hits were evaluated. The N2b component (latency range: 250–430 ms, from visual stimulus onset) and P3b component (latency range: 350–700 ms, from visual stimulus onset) of the ERPs were also evaluated. The amplitudes (in microvolts, from the maximum peak to the baseline) were measured at Fz, Cz, and Pz, and the latencies (in milliseconds, from visual stimulus onset to the maximum peak) at Cz, C3, and C4 for N2b, and at Pz, P3, and P4 for P3b, were measured.

The amplitudes (in microvolts, from the maximum peak to the baseline) and the onset latency (in milliseconds) for sLRP (from visual stimulus onset to sLRP onset) and rLRP (from rLRP onset to the button being pressed) were also measured. The onset of LRP in each category was measured using the segmented regression method developed by Schwarzenau et al. (1998). The amplitudes were measured in the 300–600 ms interval for the sLRP, and in the −200 to 0 ms interval for LRP.

### Statistical analysis

With the aim of evaluating the effect of involuntary capture of attention and the aging effect (and their interaction) on the RTs, percentage of hits, N2b and P3b latencies at midline (Cz for N2b and Pz for P3b), and on the onset latency and amplitude of sLRP and rLRP, in all three age groups, we performed two-factor analysis of variance (ANOVAs), with a between-subject factor *Group* (with three levels: young, middle-aged, and old), and a within-subject factor *Condition* (with two levels: standard and novel). We also performed three-factor ANOVAs, with a between-subject factor *Group*, and two within-subjects factors: *Condition* and *Hemisphere* (with two levels: left, right), for N2b and P3b latencies measured at lateral locations (C3 and C4 for N2b, P3, and P4 for P3b). Statistical tests were not used to evaluate the response omissions, which accounted for less than 2% of the responses.

With the aim of evaluating the effect of involuntary capture of attention and the aging effect (and their interaction) on the N2b and P3b amplitudes, in all three age groups, we performed three-factor ANOVAs, with a between-subject factor *Group*, and two within-subject factors: *Condition* and *Electrode Position*, with three levels (Fz, Cz, and Pz).

Whenever the ANOVAs revealed significant effects due to the factors or their interactions, posterior comparison of the mean values was carried out (adjusted to Bonferroni correction). Differences in results were considered significant at *p* ≤ 0.05. Greenhouse–Geisser corrections to the degrees of freedom were applied in all cases in which the condition of sphericity was not met. In these cases, the original degrees of freedom are presented together with the corrected *p-* and ε-values. All statistical analyses were performed with IBM SPSS Statistics package v.19 for Windows.

## Results

Mean values and standard deviations for the latencies and amplitudes of the N2b and P3b components, and for the onset latencies and amplitudes of the sLRP and rLRP, in the three groups of participants (Young, Middle-aged, and Old) are shown in Table 1. The *F*-values for (1) the three-factor ANOVAs (Electrode Position × Condition × Group) for N2b and P3b amplitudes at midline, (2) the two-factor ANOVAs (Condition × Group) for N2b and P3b latencies at midline, and amplitudes and onset latencies of sLRP and rLRP, and (3) the three-factor ANOVAs (Condition × Hemisphere × Group) for the N2b and P3b latencies at lateral electrodes are illustrated in Table 2.

**Table 1:** **Mean amplitudes and latencies (with SDs in parenthesis) for N2b and P3b components, and amplitudes and onset latencies for sLRP and rLRP, for each age group (Young, Middle-age, Old) in each condition (Novel, Standard)**.

		**Amplitude young**		**Amplitude middle-aged**		**Amplitude old**		**Latency/Onset young**		**Latency/Onset middle-aged**		**Latency/Onset old**	
		**N**	**S**	**N**	**S**	**N**	**S**	**N**	**S**	**N**	**S**	**N**	**S**
N2b	Fz	−7.3 (4.7)	−6.6 (3.8)	−6.3 (4.4)	−5.5 (3.8)	−6.1 (4.6)	−5.9 (4.8)	–	–	–	–	–	–
	C3	–	–	–	–	–	–	214 (54.1)	234 (51.2)	338 (76.5)	305 (44.9)	346 (64.4)	318 (48.3)
	Cz	−5.5 (6.7)	−6.5 (5.6)	−12.5 (4.8)	−10.9 (4.5)	−9.2 (7.3)	−9.4 (7.0)	257 (32.1)	259 (18.8)	339 (70.8)	318 (43.9)	325 (43.3)	307 (45.4)
	C4	–	–	–	–	–	–	226 (47.2)	235 (49.5)	353 (74.9)	308 (45.9)	349 (66.7)	312 (46.8)
	Pz	−2.8 (7.1)	0.8 (6.0)	−8.7 (4.9)	−7.5 (3.7)	−5.0 (6.0)	−5.8 (5.9)	–	–	–	–	–	–
P3b	Fz	5.1 (5.8)	5.6 (5.2)	7.0 (4.7)	6.2 (3.9)	7.4 (3.3)	6.2 (3.6)	–	–	–	–	–	–
	Cz	10.2 (6.3)	9.8 (6.6)	4.4 (6.3)	3.4 (6.2)	7.9 (5.6)	5.5 (6.1)	–	–	–	–	–	–
	P3	–	–	–	–	–	–	425 (58.3)	419 (52.6)	568 (98.9)	556 (104.6)	551 (113.5)	528 (68.3)
	Pz	19.2 (8.5)	17.0 (8.0)	8.1 (5.5)	6.6 (5.6)	11.1 (5.9)	7.8 (6.4)	433 (51.4)	421 (41.9)	580 (87.7)	516 (86.5)	557 (96.9)	503 (76.2)
	P4	–	–	–	–	–	–	421 (58.3)	417 (64.2)	570 (86.3)	522 (172.1)	569 (119.7)	517 (83.1)
sLRP	–	−4.0 (1.2)	−3.6 (1.4)	−4.5 (1.7)	−4.1 (1.3)	−4.2 (1.2)	−3.8 (1.1)	261 (71.4)	246 (51.1)	319 (45.6)	286 (36.0)	349 (62.8)	312 (58.8)
rLRP	–	−3.9 (2.0)	−3.7 (2.0)	−4.8 (1.6)	−4.1 (1.3)	−5.0 (2.4)	−4.1 (1.7)	−173 (56.4)	−180 (30.7)	−231 (64.3)	−262 (59.0)	−253 (52.0)	−260 (46.4)

N, Novel condition; S, Standard condition.

**Table 2:** ***F*-values from: three-factor ANOVAs (Electrode Position × Condition × Group) for N2b and P3b amplitudes at midline, two-factor ANOVAs (Condition × Group) for N2b and P3b latencies at midline and values for sLRP and rLPR amplitudes and onsets, and three-factor ANOVAs (Condition × Hemisphere × Group) for N2b and P3b latencies at lateral locations**.

**Amplitude, ANOVA (EP × C × G)/(C × G)**	**N2b**	**P3b**	**sLRP**	**rLRP**
EP	50.7^**^ ε = 0.8 df: 2/116	47.5^**^ ε = 0.8 df: 2/112	–	–
C	<0.1 df: 1/58	14.4^**^ df: 1/56	11.6^**^ df: 1/66	14.1^**^ df: 1/58
G	6.2^**^ df: 2/58	5.7 ^**^ df: 2/56	0.7 df: 2/66	1.0 df: 2/58
EP × C	6.8^**^ ε = 0.8 df: 2/116	20.7^**^ ε = 0.7 df: 2/112	–	–
EP × G	22.5^**^ df: 4/116	21.2^**^ df: 4/112	–	–
C × G	2.5 df: 2/58	1.7 df: 2/56	<0.1 df: 2/66	1.4 df: 2/58
EP × C × G	5.7^**^ df: 4/116	2.7^*^ df: 4/112	–	–
**LATENCY/ONSET, ANOVA (C × G)**				
C	3.9^*^ df: 1/62	15.0^**^ df: 1/61	26.3^**^ df: 1/61	5.1^*^ df: 1/55
G	22.0^**^ df: 2/62	25.1^**^ df: 2/61	11.8^**^ df: 2/61	16.3^**^ df: 2/55
C × G	1.3 df: 2/62	2.1 df: 2/61	1.4 df: 2/61	1.4 df: 2/55
**LATENCY/ONSET, ANOVA (C × H × G)**				
C	5.5^*^ df: 1/61	3.7^*^ df: 1/62	–	–
H	4.6^*^ df: 1/61	0.3 df: 1/62	–	–
G	35.4^**^ df: 2/61	27.5^**^ df: 2/62	–	–
C × H	6.4^*^ df: 1/61	1.5 df: 1/62	–	–
C × G	4.4^*^ df: 2/61	0.6 df: 2/62	–	–
H × G	2.3 df: 2/61	0.4 df: 2/62	–	–
C × H × G	<0.1 df: 2/61	0.5 df: 2/62	–	–

** p ≤ 0.01,

* p ≤ 0.05.

C, Condition factor; EP, Electrode Position factor; G, Group factor; H, Hemisphere factor; df, degrees of freedom; ε, epsilon value.

### Performance

**Figure 1:**
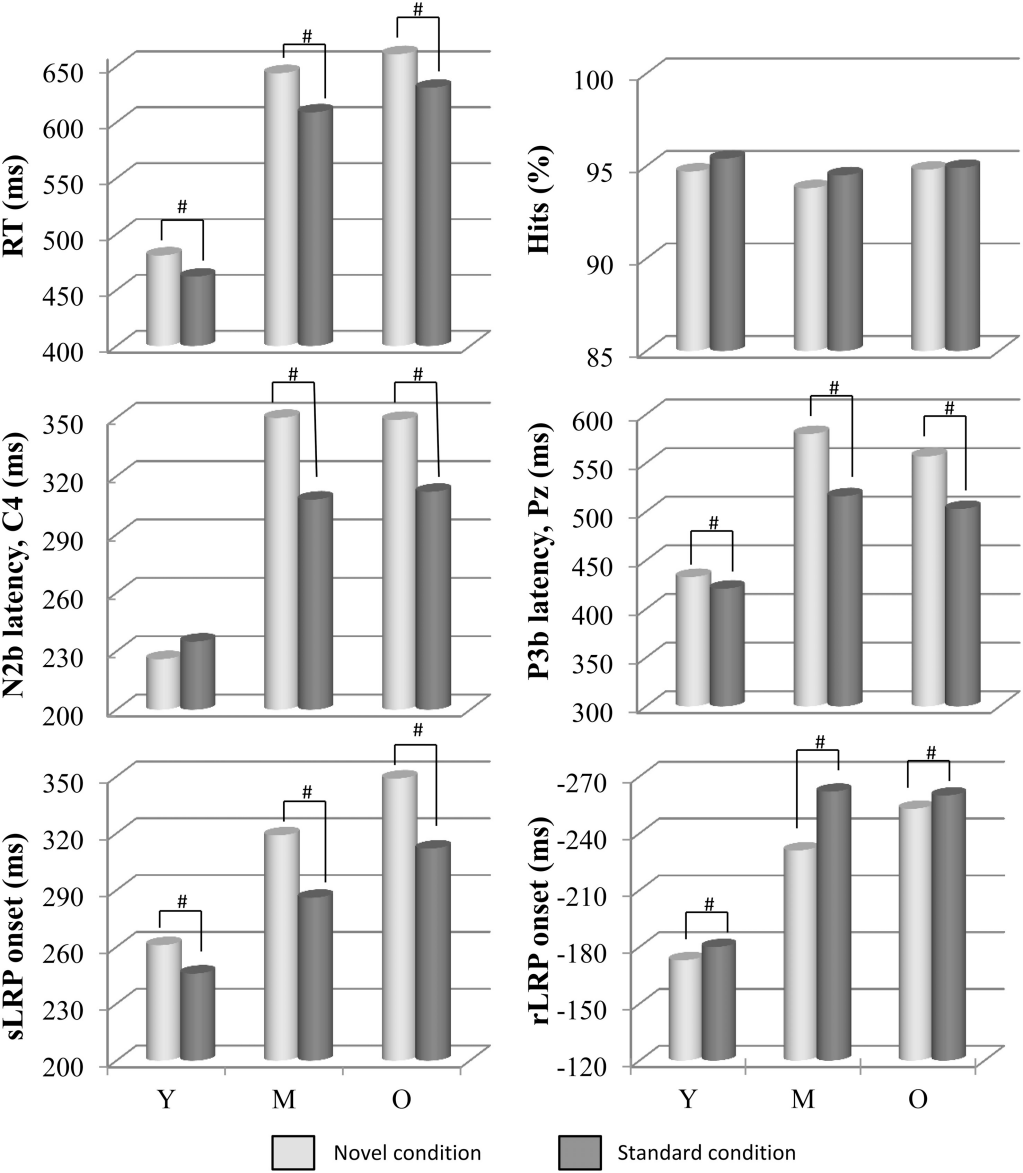
**Mean values of RT, N2b (at C4) and P3b (at Pz) latencies, sLRP and rLRP onset latencies (in ms), and percentage of hits, in each condition (novel and standard) and in each age group (Y, young; M, middle-aged; and O, old)**. Distraction effect caused by novel stimuli, respect to standard stimuli, was associated with longer RT, N2b, and P3b latencies, and sLRP onset latency. For N2b latency at C4 and C3 this distraction effect can be seen in the Middle-aged and Old groups, but not in the Young group. A facilitation effect on rLRP onset latency was also observed in the novel condition. Besides, age effects can also be seen here in longer RTs, and in every ERP component, with longer latencies for the Middle-aged and Old groups than for the Young group. #: significant difference.

The two-factor ANOVA (Condition × Group) revealed a significant effect of the *Condition* factor on the RT [*F*_(1, 73)_ = 85.0, *p* ≤ 0.0001], which was significantly longer in the novel condition (603 ms, SD: 113.1) than in the standard condition (574 ms, SD: 102.1; see Figure 1). The *Group* factor was also significant [*F*_(2, 73)_ = 36.7, *p* ≤ 0.0001], as the RT was significantly longer in the Old (646 ms, SD: 85.1) and Middle-aged groups (627 ms, SD: 84.6) than in the Young group (472 ms, SD: 54.9).

The two-factor ANOVA (Condition × Group) did not reveal any significant effect of the factors or their interaction (*p* > 0.05) on the percentage of hits (see Figure 1).

### ERPs

#### Visual stimuli processing: N2b and P3b

**Figure 2:**
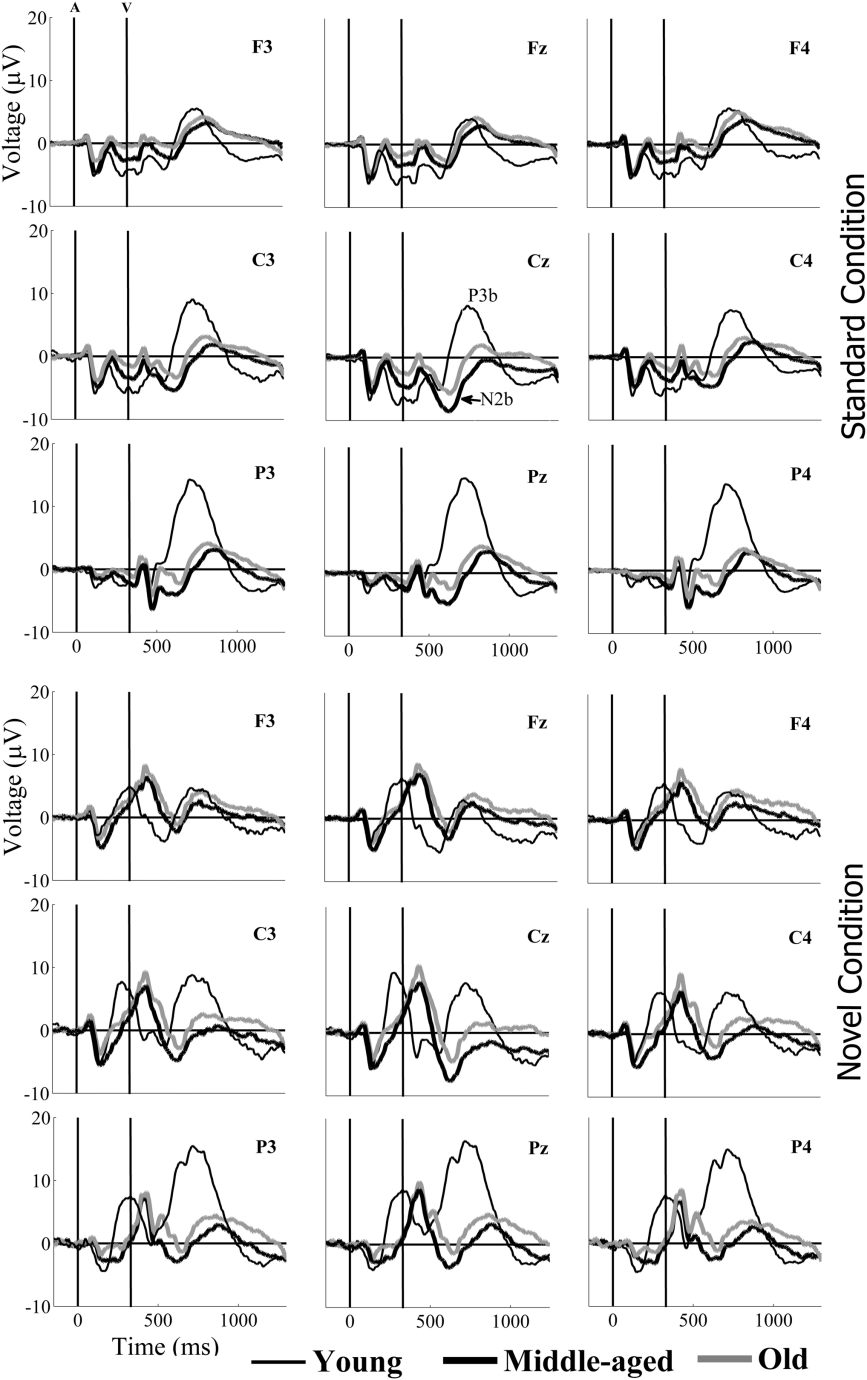
**Grand-average event-related potential waveforms, for the Young (thin black line), Middle-aged (thick black line), and Old (thick gray line) groups, in the standard (upper pannel) and the novel (lower pannel) conditions, at F3, Fz, F4, C3, Cz, C4, P3, Pz, and P4 electrode sites**. At parietal locations (in both conditions), the N2b amplitude was larger in the old and middle-aged than in the young participants. At parietal and central locations, P3b amplitudes were larger in the young than in the old and middle-aged participants.

The mean latency of the N2b component was 311 ms (SD: 53.5) at the Cz electrode site (where the maximum peak amplitude was recorded at midline, see Table 1 and Figure 2). The two-factor ANOVA (Condition × Group) for the N2b latency at Cz revealed a significant effect of the *Condition* factor, as this parameter was significantly longer in the novel condition than in the standard condition (see Figure 1). The ANOVA also revealed a significant effect of the *Group* factor, as N2b latency was significantly longer in the Middle-aged and Old groups than in the Young group.

The three-factor ANOVA (Condition × Hemisphere × Group) for the N2b latency revealed significant main effects of *Condition* factor and *Hemisphere* factor, and a significant *Condition x Hemisphere* interaction, as N2b latency was significantly longer in the novel than in the standard condition at the right hemisphere, and it was significantly longer in the right than in the left hemisphere in the novel condition (see Table 1). Besides, a significant *Condition* × *Group* interaction was obtained, as N2b latency was significantly longer in the novel than in the standard condition in the Middle-aged and Old groups, but it did not show significant differences between conditions in the Young group (see Figure 1); and N2b latency was significantly longer in the Middle-aged and Old groups than in the Young group in both novel and standard conditions (see Table 1).

For the N2b amplitude, the three-factor ANOVA (Electrode Position × Condition × Group) revealed significant effects of *Electrode Position* factor, *Group* factor, and *Electrode Position* × *Group*, *Electrode Position* × *Condition*, and *Electrode Position* × *Condition* × *Group* interactions. The N2b amplitude was significantly larger in the novel than in the standard condition at Cz in the Middle-aged group and at Pz in the Young group (see Table 1). At the Cz electrode site, in both the novel and standard conditions, the N2b amplitude was significantly larger for the Middle-aged group than for the Young group, while at the Pz, it was significantly larger for the Middle-aged and Old groups than for the Young group (see Figure 2). In both conditions, the N2b amplitude was significantly larger at the Cz than at Pz and Fz electrodes in the Middle-aged and the Old groups, while in the Young group it was significantly larger at Fz and Cz than at Pz (see Table 1).

The mean latency of the P3b component was 507 ms (SD: 91.0) at the Pz electrode site (where the maximum peak amplitude was recorded at midline; see Table 1 and Figure 2). The two-factor ANOVA (Condition × Group) for the P3b latency at Pz revealed a significant effect of the *Condition* factor, which was significantly longer in the novel condition than in the standard condition (see Figure 1). The ANOVA also revealed a significant effect of the *Group* factor, as the P3b latency was significantly longer in the Middle-aged and Old groups than in the Young group. The three-factor ANOVA (Condition × Hemisphere × Group) for the P3b latency at P3 and P4 electrodes revealed the same effects *Group* and *Condition* (see Figure 2).

For the P3b amplitude, the three-factor ANOVA (Electrode Position × Condition × Group) revealed a significant main effect of each factor, and of the *Electrode Position × Group*, *Electrode Position × Condition*, and *Electrode Position × Condition × Group* interactions. P3b amplitude was significantly larger in the novel than in the standard condition at Pz for the three age groups, and also at Cz for the old group (see Table 1). In both conditions, this parameter was significantly larger in the Young group than in the Middle-aged group at Cz, and it was significantly larger in the Young than in the Middle-aged and the Old groups at Pz (see Figure 2). In the Young group, the P3b amplitude was significantly larger at Pz than at Cz and Fz, and at Cz than at Fz; in the Middle-aged group, it was significantly larger at Pz and Fz than at Cz; and in the Old group, this parameter was significantly larger at Pz than at Cz (Table 1).

#### Response preparation and selection: sLRP and rLRP

**Figure 3:**
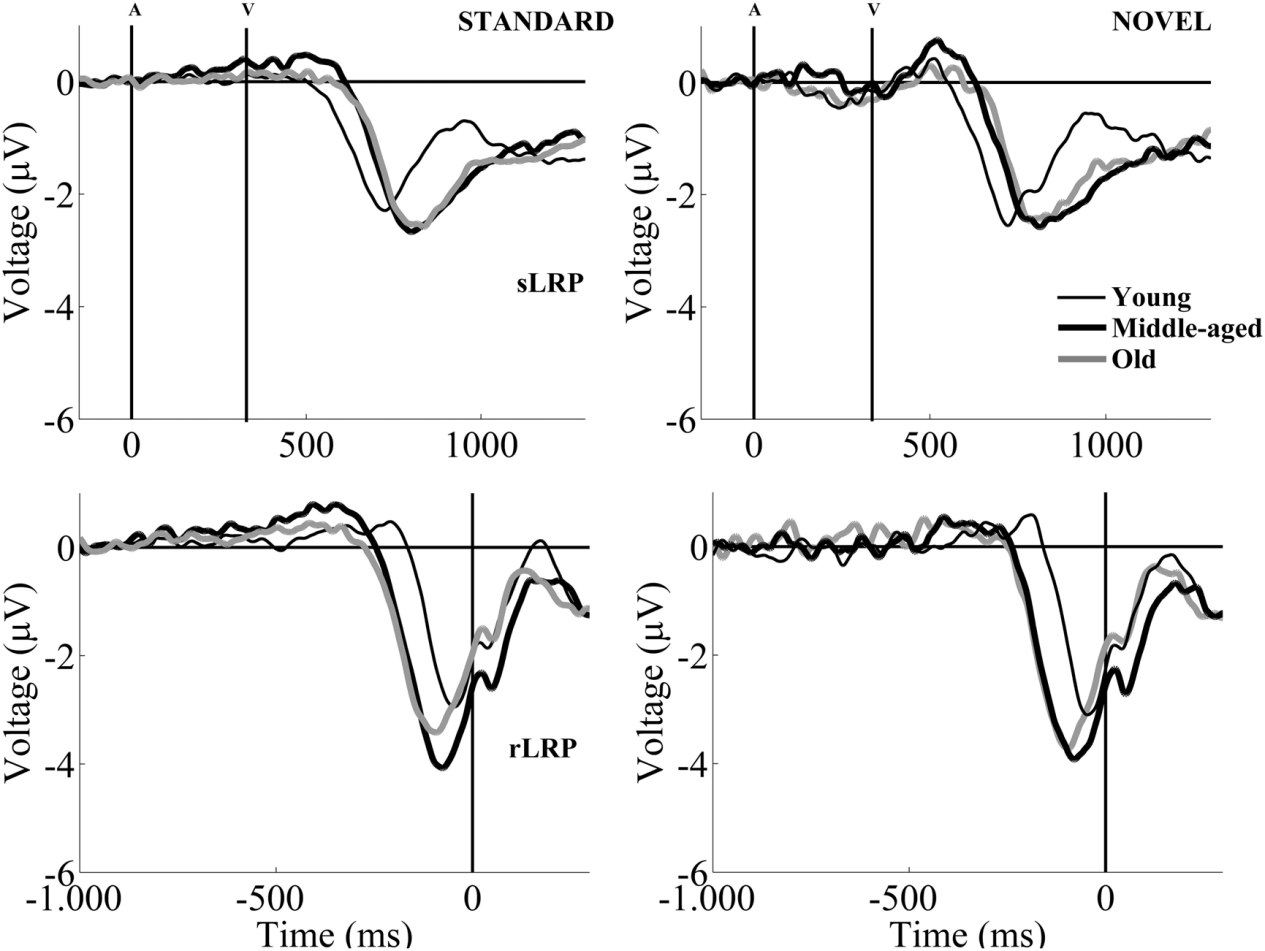
**Grand-average sLRP (upper figures) and rLRP waveforms (lower figures) for the Young (thin black line), Middle-aged (thick black line), and Old (thick gray line) groups, in the standard (left) and the novel (right) conditions**. In order to obtain the sLRP and rLRP waveforms, the differences between contralateral and ipsilateral activation for C3 and C4 electrode pairs in each hemisphere were calculated. The differences were then averaged (Gratton et al., 1988). The method can be summarized by the following formula: [[(C4–C3)_left hand movements_ + (C3–C4)_right hand movements_]/2].

The mean onset latency for the sLRP (from the visual stimulus onset to sLRP onset) was 305 ms (SD: 67.8; see Table 1 and Figure 3). For the sLRP onset latency, the two-factor ANOVA (Condition × Group) revealed a significant effect of the *Condition* factor, as the sLRP onset latency was significantly longer in the novel than in the standard condition (see Figure 1). The ANOVA also revealed a significant effect of the *Group* factor, as the sLRP onset latency was significantly longer in the Middle-aged and Old groups than in the Young group (see Figure 3).

The two-factor ANOVA (Condition × Group) for the sLRP amplitude revealed a significant effect of the *Condition* factor, as the amplitude was significantly larger in the novel than in the standard condition (see Table 1).

The mean onset latency for the rLRP was −227 ms (SD: 51.5; see Table 1 and Figure 3). The two-factor ANOVA (Condition × Group) for the rLRP onset latency revealed a significant main effect of the *Condition* factor, as this parameter was significantly earlier relative to the response in the standard than in the novel condition, maybe reflecting a facilitation effect in motor preparation in the latter. The ANOVA also revealed a significant effect of the *Group* factor, as the latency was earlier in the Middle-aged and Old groups than in the Young group.

For the rLRP amplitude, the two-factor ANOVA (Condition × Group) revealed a significant main effect of the *Condition* factor, as the rLRP amplitude was significantly larger in the novel than in the standard condition.

## Discussion

### Performance

There were no differences between conditions for the percentage of hits, in accordance with other studies (Escera et al., 1998, 2001; Andrés et al., 2006). Longer RTs were observed in the novel than in the standard condition, as also found in previous studies using a similar auditory-visual distraction-attention task reporting delayed RTs to target stimuli caused by preceding irrelevant novel sounds (Escera et al., 1998, 2001; Andrés et al., 2006; Parmentier and Andrés, 2010). So, RTs appear to be a suitable parameter for assessing the distraction produced by the involuntary capture of attention on the target stimuli processing (Escera et al., 1998, 2001).

Nevertheless, this distraction effect was not greater in aging as in Andrés et al. (2006), replicating the results in Parmentier and Andrés (2010). Both studies used the same task design, and both young and old groups had very similar mean ages (Andrés et al., 22.2 and 68 years old, respectively; Parmentier and Andrés, 21.8 and 68.8 years old, respectively). So, the inconsistency in the increase of the distraction effect among studies could only be explained by the interindividual differences among participants.

On the other hand, the RTs were longer in both groups of older participants (Old and Middle-aged) than in the Young group, as also found in several studies demonstrating an age-related increase in RTs in a variety of cognitive tasks (see Salthouse, 2000).

### Latency effects

N2b and P3b latencies, and sLRP onset latency, were longer in the novel than in the standard condition. This may indicate that the non-attended novel stimulus eventually captures attention provoking a distraction effect reflected in a slowing down of target evaluation and categorization processes in WM, as well as of the response selection processes. This distraction effect was not modulated by aging for P3b or sLRP. Nevertheless, the N2b latency was significantly longer in the novel than in the standard condition only in the Old and Middle-aged groups, at lateral electrodes (C3 and C4) (see Table 1). So, the unattended novel stimulation affected the active evaluation of the target stimulus in WM in middle-aged and old participants delaying this process, but it was not observed in young adults.

Interestingly, the rLRP onset latency was earlier in the standard condition than in the novel condition in all three age groups. This may indicate that the unattended novel stimulus caused some sort of facilitation effect that resulted in a reduction of the time needed to plan and execute the motor response.

With regard to the Group effect, N2b and P3b latencies were longer in the Middle-aged and Old groups (with no differences between them) than in the Young group, in accordance with previous studies using oddball or Go/NoGo tasks (Amenedo and Díaz, 1998a,b; Czigler et al., 2006; Gaál et al., 2007; Schiff et al., 2008; Ashford et al., 2011; Schmiedt-Fehr and Basar-Eroglu, 2011; Juckel et al., 2012). Lower speed of information processing is one of the hallmarks of cognitive aging (Van Deursen et al., 2009), and our results specifically show an age-related slowing in the evaluation and the categorization of the target stimuli from middle age onwards.

The sLRP onset latency showed the same age-related effect. This is consistent with previous studies that used different tasks (Wild-Wall et al., 2008; Cespón et al., 2013) although other researchers did not find such differences (Yordanova et al., 2004; Kolev et al., 2006; Roggeveen et al., 2007). The time of preparation of the response (indexed by the rLRP) was longer in the Middle-aged and Old groups (as the onset latency occurred earlier, with no differences between them) than in the Young group, as in previous studies (Yordanova et al., 2004; Roggeveen et al., 2007; Wild-Wall et al., 2008; Cespón et al., 2013). Hence, these results provide additional support to the idea that age-related slowing affects both the selection and preparation of the motor response. In the case of the sLRP, this may be due to slower transmission of information from visual motor areas (Wild-Wall et al., 2008). The rLRP result, may be due to either the need for a longer activation of the motor cortex in old and middle-aged participants to enable response execution (Kolev et al., 2006; Cespón et al., 2013), or to an age-related strategy to emphasize response accuracy (Osman et al., 2000).

### Amplitude effects

The N2b (in Young and Middle-aged), P3b, sLRP, and rLRP (in the three age groups) amplitudes were larger in the novel than in the standard condition. In the Escera et al.'s (1998) study the authors observed for young people similar results for N2b and P3b amplitudes, accompanied by longer RTs in the novel than in the standard condition (as an index of the distraction effect). On the other hand, SanMiguel et al. (2010) also found larger P3b amplitudes, but with shorter RTs, in the novel condition than in the standard condition. These authors interpreted their results as indexes of a facilitation effect produced by the novel stimulation.

We consider that the larger amplitudes of the ERP components evaluated in the novel than in the standard condition may indicate that the novel stimuli acted as activating signals, causing an enhanced arousal (Polich and Kok, 1995; Ashford et al., 2011). The larger amplitude obtained in the novel condition may reflect the response of the neuromodulatory locus coeruleus-norepinephrine (LC-NE) system in information processing, i.e., potentiation of the response to motivationally significant events (Nieuwenhuis et al., 2005), which may also affect the N2b and LRP amplitudes. There is some evidence that the LC-NE system is involved in motor control (Benarroch, 2009). The anterior cingulate and the dorsolateral prefrontal cortices, proposed as N2b generators (Potts and Tucker, 2001; Folstein and Van Petten, 2008), seem to be connected up to the LC, linking circuits involved in cognitive processing with the LC-NE system (Aston-Jones and Cohen, 2005).

Interestingly, the old group did not show larger N2b amplitude in the novel than in the standard condition. Noradrenergic function seems to be enhanced in older relative to young adults (Elrod et al., 1997; Raskind et al., 1999), which may mask the differences between both conditions (novel vs. standard) in this age group.

With regard to the aging effect, the N2b amplitude was larger in the middle-aged and old participants than in the young participants, which is also consistent with previous findings (Friedman et al., 1993; Iragui et al., 1993; Anderer et al., 1996; Czigler et al., 2006; Schmiedt-Fehr and Basar-Eroglu, 2011). Given that the number of correct responses did not discriminate among groups, this may indicate that older people must assign more attentional resources to the evaluation of target stimuli than young participants, probably as compensatory mechanism for correct performance. Moreover, the N2b amplitude did not differentiate between the middle-aged and old participants, and was maximal at central locations in the Middle-aged and Old groups, whereas it showed a more frontal distribution in the Young group. Some authors have reported age-related amplitude reductions at anterior scalp areas (Enoki et al., 1993; Iragui et al., 1993; Anderer et al., 1996), or a change to a more posterior scalp distribution (Friedman et al., 1993). Our findings support age-related changes in neural networks facilitating enhanced allocation of processing resources for evaluation of relevant stimuli in WM. These changes appear to begin relatively early in middle age and remains fairly stable from 50 onwards.

The P3b amplitude was larger in the Young than in the Middle-aged and Old groups, at parietal and central locations, which is also consistent with previous findings (Amenedo and Díaz, 1998a; Czigler et al., 2006; Hämmerer et al., 2010; Ashford et al., 2011; Schmiedt-Fehr and Basar-Eroglu, 2011; Juckel et al., 2012; O'Connell et al., 2012). In the Young group, a graded distribution pattern was observed for the P3b amplitude (Pz > Cz > Fz), in consonance with previous reports (Kutas et al., 1994; Czigler et al., 2006; Gaál et al., 2007). In the Middle-aged and Old groups, P3b amplitude distribution was more homogeneous across electrode sites (Kutas et al., 1994; Amenedo and Díaz, 1998a; Anderer et al., 2003; Cid-Fernández et al., 2014), which may reflect the need to engage frontal structures related to WM (Fabiani and Friedman, 1995) processing.

In Middle-aged and Old groups, the relative under-recruitment of task-related brain networks (Schmiedt-Fehr and Basar-Eroglu, 2011), possibly due to a decline in the activity of the posterior cortex (Amenedo and Díaz, 1998a; Schiff et al., 2008; Ashford et al., 2011) and also to a decline in cholinergic neurotransmission (Schiff et al., 2008; Schmiedt-Fehr and Basar-Eroglu, 2011), seem to be accompanied by an over-recruitment of frontal networks. This may reflect the need to engage, as compensatory mechanism, frontal structures related to WM processing (Fabiani and Friedman, 1995), in accordance with the well-known Posterior-Anterior Shift in Aging model (PASA; Davis et al., 2008).

## Conclusions

Aging was associated with slower reaction times, as well as slowing of target stimulus processing (longer N2b and P3b latencies) and the associated selection and preparation of the corresponding motor response (longer sLRP and rLRP onset latencies).

The involuntary capture of attention triggered by novel irrelevant auditory stimuli relative to the standard irrelevant auditory stimuli was associated with a distraction effect in all three age groups under study (Young, Middle-aged and Old), with longer RT, longer time of stimulus categorization in WM (longer P3b latencies), and longer time for selection of the motor response (longer sLRP onset latency). A facilitation effect on the response preparation to the target (earlier rLRP onset latency) and an increase in the global arousal (larger amplitudes in all ERP components evaluated, except for N2b amplitude in the Old group) were also observed in the novel condition.

The distraction effect was also found in both older groups (Middle-aged and Old) regarding stimulus evaluation processes in WM (longer N2b latency in novel condition than in standard condition), but it was not observed for the Young group. This result reflects an age-related modulation of the distraction effect on the evaluation of target stimuli in WM, with a slowing of evaluation process that seem to affect people from 50 years onwards, without differences between middle-aged and older adults.

## Disclosure statements

All participants gave their written informed consent prior to participation in the study. The research project was approved by the Galician Clinical Research Ethics Committee (Xunta de Galicia, Spain). The study was performed in accordance with the ethical standards established in the 1964 Declaration of Helsinki (Lynöe et al., 1991).

### Conflict of interest statement

The authors declare that the research was conducted in the absence of any commercial or financial relationships that could be construed as a potential conflict of interest.
